# The socio-spatial determinants of COVID-19 diffusion: the impact of globalisation, settlement characteristics and population

**DOI:** 10.1186/s12992-021-00707-2

**Published:** 2021-05-20

**Authors:** Thomas Sigler, Sirat Mahmuda, Anthony Kimpton, Julia Loginova, Pia Wohland, Elin Charles-Edwards, Jonathan Corcoran

**Affiliations:** Queensland Centre for Population Research, The University of Queensland, St Lucia, Queensland 4072 Australia

**Keywords:** COVID-19, Coronavirus, Spatial diffusion, Globalisation, Urbanisation, Quantile regression

## Abstract

**Background:**

COVID-19 is an emergent infectious disease that has spread geographically to become a global pandemic. While much research focuses on the epidemiological and virological aspects of COVID-19 transmission, there remains an important gap in knowledge regarding the drivers of geographical diffusion between places, in particular at the global scale. Here, we use quantile regression to model the roles of globalisation, human settlement and population characteristics as socio-spatial determinants of reported COVID-19 diffusion over a six-week period in March and April 2020. Our exploratory analysis is based on reported COVID-19 data published by Johns Hopkins University which, despite its limitations, serves as the best repository of reported COVID-19 cases across nations.

**Results:**

The quantile regression model suggests that globalisation, settlement, and population characteristics related to high human mobility and interaction predict reported disease diffusion. Human development level (HDI) and total population predict COVID-19 diffusion in countries with a high number of total reported cases (per million) whereas larger household size, older populations, and globalisation tied to human interaction predict COVID-19 diffusion in countries with a low number of total reported cases (per million). Population density, and population characteristics such as total population, older populations, and household size are strong predictors in early weeks but have a muted impact over time on reported COVID-19 diffusion. In contrast, the impacts of interpersonal and trade globalisation are enhanced over time, indicating that human mobility may best explain sustained disease diffusion.

**Conclusions:**

Model results confirm that globalisation, settlement and population characteristics, and variables tied to high human mobility lead to greater reported disease diffusion. These outcomes serve to inform suppression strategies, particularly as they are related to anticipated relocation diffusion from more- to less-developed countries and regions, and hierarchical diffusion from countries with higher population and density. It is likely that many of these processes are replicated at smaller geographical scales both within countries and within regions. Epidemiological strategies must therefore be tailored according to human mobility patterns, as well as countries’ settlement and population characteristics. We suggest that limiting human mobility to the greatest extent practical will best restrain COVID-19 diffusion, which in the absence of widespread vaccination may be one of the best lines of epidemiological defense.

**Supplementary Information:**

The online version contains supplementary material available at 10.1186/s12992-021-00707-2.

## Introduction

The Coronavirus disease (COVID-19) has spread more globally and rapidly than previous outbreaks (e.g., the 1918 Spanish Influenza pandemic and the 2003 SARS epidemic) [1], which suggests that rising international connectivity [2, 3] and urbanisation [4, 5] have played a key role in its diffusion *between* and *within* territories. In the pandemic’s early stages, countries with high numbers of reported cases (e.g. Italy, Spain, the United Kingdom and the United States) and high numbers of reported cases per capita (e.g. Qatar, Luxembourg, Panama and Bahrain) have been highly globalised nations with high levels of urbanisation and human mobility, whilst those with fewer cases are mostly less globalised, with smaller numbers of visitors, lower rates of urbanisation, and in general less domestic mobility [6]. This observation holds true at the national scale as well, in that major outbreaks of COVID-19 were reported in the pandemic’s early stages in countries’ densest, and often most globalized and affluent, regions. For example, Lombardia (Italy) [7, 8], New York (the United States) [9], Madrid (Spain) [10], and Tehran (Iran) [11] all by far outnumbered cases in other regions within their respective countries in the first few months of 2020.

This exploratory study seeks to test the role played by globalisation, settlement and population characteristics to explain the spatial diffusion of reported COVID-19 cases at a global scale in the early stages of the pandemic. Widely understood to have diffused geographically from a single point of origin in China in late December 2019 [12, 13], spatial diffusion across country borders was at first relatively slow. It took 45 days for the virus to spread to 30 countries, areas or territories [14]. After this time, geographical diffusion accelerated and within the next 45 days, COVID-19 would reach nearly all global territories [14]. By April 8th 2020 – the final week in this study – there had been 20,277,716 reported cases recorded within the COVID-19 Data Repository by the Center for Systems Science and Engineering at Johns Hopkins University (JHU) [15]. Only 12 states and territories had purportedly remained free of COVID-19 by the end of May 2020, including 10 small and isolated Pacific island states, and two countries relatively closed to outside influence: Turkmenistan and North Korea [16].

Despite extensive epidemiological research and mathematical modelling of the COVID-19 transmission [7, 17–22], there has been a lacuna of work aiming to understand how social and geographic factors converge to explain COVID-19 diffusion on a global scale. In this exploratory paper, we redress this deficit though empirically demonstrating how globalisation, and the human settlement and population characteristics of countries explain the spatio-temporal diffusion patterns of reported COVID-19 cases, and how this relationship shifted early on in the pandemic (Weeks 10–15), when travel restrictions were still relatively incipient yet viral transmission began to globalise rapidly.

## Background

Infectious diseases diffuse over space and time through inherently geographical processes [23]. The geographical concept of spatial diffusion is defined as the spread of a phenomenon across space [24], of which disease diffusion through interpersonal transmission is but one variant [23, 25]. Here, we investigate the role of globalisation, settlement and population characteristics as socio-spatial determinants of reported COVID-19 diffusion between countries as an outcome of transmission between individuals. Although each new case is by definition a product of interpersonal transmission—both directly via contact, and indirectly via fomites—diffusion can occur across large distances as an outcome of human movement and mobility. Understandings of viral transmission lie more firmly within the academic domain of virology than diffusion does, which is a fundamentally geographic phenomenon that can be applied to many other forms of spread (for example, innovation diffusion [24]). Different underlying processes characterise types of spatial diffusion [26, 27]. *Expansion diffusion* identifies the general tendency for phenomena to spread ‘outward’, and infectious diseases are most associated with *contagious (expansion) diffusion*, indicating direct transmission between neighbours due to their physical proximity.

As infectious diseases spread through the global population, different types of diffusion come into play, often in combination [25, 27]. Disease spread that occurs over a large distance from its origin is captured by *relocation diffusion*, which is often mobilised by air travel or other modes of extra-local transportation. On a global scale, mobility and connectivity between countries collectively contribute to disease outbreaks across the globe, an observation brought forward by previous research on human rhinovirus, influenza, and SARS [28, 29]. Indeed, globalisation in its diverse forms is diminishing the role of physical (Euclidian) distance in diffusion. Though disease vectors require human contact, the speed and ubiquity of global transportation and travel have led to time-space compression [2, 30], which reduces the time-distance required to connect any two global points.

In recent studies [31, 32], globalisation has been shown to be positively linked to the reported numbers of COVID-19 cases in that more globalised countries experience higher exposure to outbreaks [32, 33], as do ‘global cities’ within countries [4]. On a global scale, Sirkeci and Yüceşahin [34] suggest that the spread of COVID-19 in March 2020 followed a relocation diffusion pattern, while Kuebart and Stabler [35] observe relocation diffusion of COVID-19 in Germany based on existing interpersonal networks. Internationally, globalisation supports relocation diffusion, as public health studies have repeatedly acknowledged [22, 36]. COVID-19 has rapidly spread via international air [37, 38] and sea [39] travel connecting countries with high levels of tourism and trade [40]. Another study [31] found that almost all KOF (Swiss Economic Institute) globalisation sub-indices [41] exhibit a robust, positive association with the number of COVID-19 reported cases, with social globalisation—which proxies migration and civil rights among other measures—being the most important predictor both in magnitude and statistical significance.

Another mode of spatial diffusion is through *hierarchical diffusion*, which characterises spread from large settlements to smaller ones, or from more internationally significant cities (e.g. ‘global city-regions’) to those less significant. In the case of infectious diseases, previous research suggests that large metropolitan areas experience greater spread due to the larger number of people, their closer proximity and increased movement [5, 29, 42–44]. Fortaleza et al. [45] observed hierarchical diffusion of COVID-19 from the largest cities to smaller settlements in Brazil. Similarly, Sirkeci and Yüceşahin [34] observe hierarchical diffusion of COVID-19 infection in countries including the United States, the United Kingdom, South Korea and Italy among others.

Certain settlement characteristics are associated with hierarchical diffusion, including the level of urbanisation, density and accessibility. Larger and denser cities have been shown to increase vulnerability to infectious disease spread [46] by creating the requisite preconditions for higher numbers of human interactions wherein higher densities act to increase the intensity of such interactions [47]. Andersen et al. [48] find that urbanisation is a significant predictor of COVID-19 transmission within the United States, while Carozzi [49] finds urban density to be a predictor.

Additionally, there are marked differences in population characteristics—population size, development levels, household size and age structure— affecting the spread of an infectious disease [38, 50]. We examine this using four population characteristics of individual countries: Human Development Index (HDI); population aged over 65; mean household size; and national population size. These variables have been included to control and build upon recently published findings that can explain the rates of COVID-19 outbreak at the early stages [9, 34, 51].

### Data

**Fig. 1:**
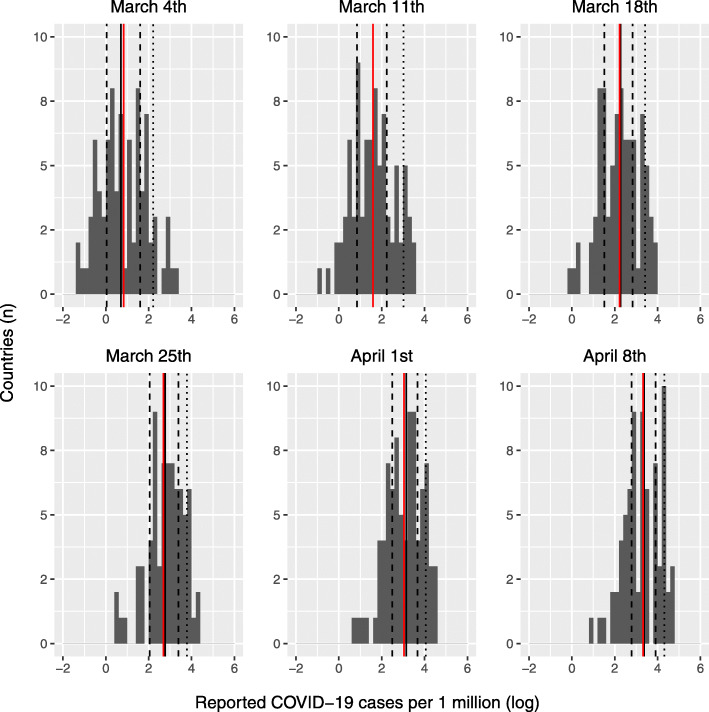
Distributions of cumulative reported COVID-19 cases per million population (log transformed). Graphs show the 10th week (ending March 4th) until the 15th week (ending April 8th) of 2020. The red line indicates the mean and the black lines quantiles

We employ quantile regression [52, 53] to examine how globalisation, settlement characteristics and population characteristics impact the cumulative total of reported COVID-19 cases per one million inhabitants over a six-week period from the 10th week (ending March 4th) until the 15th week of 2020 (ending April 8th). Figure 1 shows the distribution of cases over the study period.

The study period was chosen based on its relation to the variables applied to explain reported COVID-19 diffusion. Prior to the first week of March 2020, there were insufficient data points to study the disease on a global scale. Data collection accelerated with the number of reported cases when COVID-19 was declared a global pandemic by the World Health Organisation (WHO) on March 11th 2020 [54], just over 2 months after its outbreak. During the six-week period of the study period, the number of reported cases increased by 1433% and the number of countries and territories affected more than doubled, counting those enumerated within the Johns Hopkins University (JHU) repository [15] as extracted on May 13th 2020.

The JHU dataset remains the most globally-representative data available. Such global near real-time data were not available to scholars studying previous epidemics, with empirical studies concerned largely with the disease diffusion at the national scale. However, developers of this database recognise that drawing data from this, and similar global repositories, is not without caveats [55]. Among these, previous research and commentary have identified the ambiguity of definitions and reporting frequencies; discrepancies in reporting across sources and countries; data inconsistency and completeness [56]; and intentional misreporting [57]. For example, it has been suggested that at the end of March 2020, the average detection rate of COVID-19 was 6 %, increasing to 9 % 2 weeks later [56]. Though a register within the GitHub repository contains more than a thousand items associated with how JHU data are collected and reported [58], Stokes et al. [59] report that the JHU data are consistent with CDC data in the United States, and “still stands as the authoritative source of global COVID-19 epidemiological data”, according to Gardner et al. [60]. We align with published studies that use the JHU database [61–65], yet share the proviso that these reported COVID-19 cases are the best proxy for the true infection rate given that an unknown number of cases remain undetected. We return to a number of potential implications for our findings in the Discussion section.

**Fig. 2:**
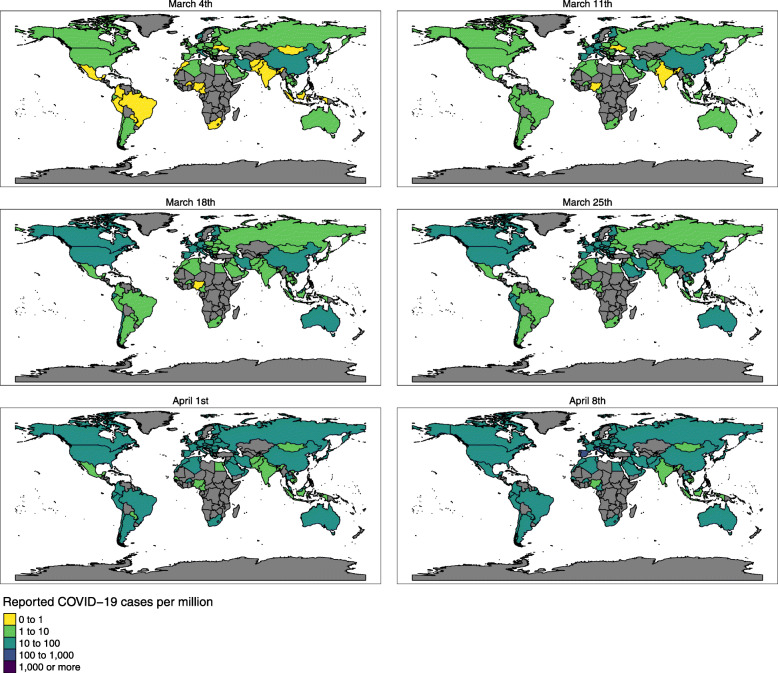
Choropleth map of reported cases of COVID 19 per million population for the 84 countries included in the analysis over weeks 10 to 15 (ending March 4th and April 8th 2020, respectively)

**Fig. 3:**
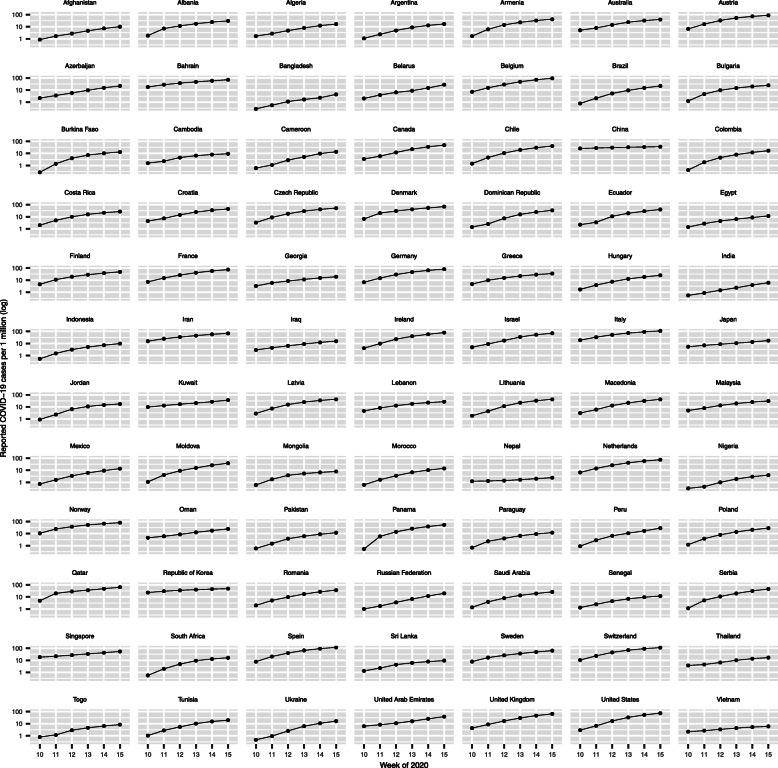
Diffusion of reported COVID-19 cases per million population (log transformed) over weeks 10–15 (ending 4th March and April 8th 2020, respectively) across 84 countries

Over the six-week study period, the number of reported COVID-19 cases spread globally to incorporate an increasing number of countries and cases. Figures 2 and 3 show the geographical (Fig. 2) and temporal diffusion (Fig. 3) of reported COVID-19 cases between February 27th and April 8th 2020.

The dependent variable in the quantile regression model is the number of cumulative total of reported COVID-19 cases per one million inhabitants (log-transformed) by country (or territory) and by week. The denominator for the dependent variable is the 2019 mid-year population by country drawn from the United Nations World Population Prospects [66]. Eighty-four countries had consistent available data for the duration of the study period and were therefore included in the model.

The choice of quantile regression allows us to go beyond the mean relationship between the response and the predictor variables to reveal statistical relationships at specific points along the distribution [52, 53, 67, 68]. In this way, we detail our discussion on how the impacts of globalisation, settlement characteristics and population characteristics on the global diffusion of reported COVID-19 cases vary across the distribution. In contrast, a mean model approach would explain how these impacts occur in general thus potentially failing to capture impacts towards the ends of the pandemic spectrum.

Although mean regression models are highly sensitive to outliers, different quantile estimations can also be influenced by outliers at different locations [69, 70]. For example at the 50th quantile in the last 3 weeks of the study, China, Iran and Japan stand out as influential observations which might have overly impacted the significance of each variable.

The quantile model includes a total of 11 independent variables to explain reported COVID-19 cases per one million inhabitants (log-transformed) by country (or territory) by week. To understand the role of globalisation in COVID-19 diffusion, we include three variables from the KOF globalisation index [41, 71, 72]: de facto interpersonal globalisation, de facto financial globalisation and de facto trade globalisation to represent globalisation. These sub-indices proxy migration, tourism and business flows, which are known to be positively associated with outbreaks of infectious diseases by exposing countries to the outside world [31, 32, 36, 43, 73–75]. Globalisation variable 1 is de facto interpersonal globalisation, which is a KOF sub-index of social globalisation that includes indicators of international traffic, transfers, international tourism, international students and migration [41]. An early study of the COVID-19 spatial diffusion [34] shows that the volume of migration flows has been a strong indicator for the international spread of the pandemic. Globalisation variable 2 is de facto trade globalisation, another KOF sub-index of economic globalisation that reflects trade in goods and services as well as trade partner diversity [41]. Globalisation variable 3 is de facto financial globalisation, a KOF sub-index of economic globalisation. It is comprised of measures of foreign direct investment, portfolio investment, international debt, international reserves, and international income payments [41].

To understand the role of settlement characteristics in reported COVID-19 diffusion, we include four variables that measure various national-scale dimensions of settlement characteristics: urbanisation rate; population density; maximum urban population density; and areal accessibility (measures the average drive time of the national population from smaller to larger settlements) [76]. These operationalise human interaction within national boundaries, with recent publications suggesting that diffusion happens more rapidly in cities that are dense, well-connected, and accessible [4, 5, 34, 46, 47]. Settlement variable 1 is urbanisation rate, defined as the proportion of a national population located in cities or metropolitan regions (national definitions vary). We selected this variable as cities are more prone to early disease diffusion than rural areas due to higher concentration of interaction and movement in urban areas [5], and documented higher sensitivity of large cities to the spread of infectious diseases [4, 29, 77]. Settlement variable 2 is population density, defined as the population per square kilometer across a national territory. Population density proxies the higher intensity of human interaction which makes disease transmission more likely. The literature shows that population density has a high impact on the outbreak of infectious diseases [47]. While a previous study [34] did not detect a relationship between population density and total reported COVID-19 cases, there is a broader literature that suggests an association between population density and the outbreak of infectious diseases [47].

Settlement Variable 3 is urban density [maximum], defined as the population per square kilometer of the densest city in a country. This variable has been selected as a proxy for the level of density exhibited in a country’s main urban areas, compensating for the fact that many countries with relatively low levels of urbanisation at a national scale (e.g. Pakistan, Bangladesh) in fact have some of the world’s highest urban densities within their cities. Settlement Variable 4 is areal accessibility, defined as an area-weighted average of driving time to locations with at least 1500 inhabitants per square km [76]. This variable has been selected based on a previous study [46] in which the authors argue that extended urbanisation may result in increased vulnerability to an infectious disease spread. Urban accessibility captures the variations in suburbanisation and peri-urbanisation across countries.

To understand the role of national population characteristics in reported COVID-19 diffusion, we employ: HDI; population age structure (65+); median household size; and population size. Research suggests that COVID-19 is more likely to spread in more-developed countries with higher levels of international migration than in countries with lower levels of development and migration [31], at least in early stages. Affluent, healthy, and educated populations (HDI) are more likely to be highly mobile. Although larger household sizes and national populations are associated with increased reporting of COVID-19 cases, these are not clear-cut relationships [9]. Older populations, or populations with higher mortality rates, are more likely to get tested than younger populations that may be asymptomatic [51, 78]. Population variable 1 is HDI (Human Development Index), which captures a holistic picture of individual countries and has been used as an indicator of the macro environment in a previous study [34] written in the early period of the pandemic. The study found that each unit increase in the HDI score is associated with five more reported COVID-19 cases. Populations in countries with higher HDI are more affluent, healthier, and better educated, meaning that their overall mobility potential would be higher. Population variable 2 is population aged 65 and over (%), which is the proportion of the population aged 65 years and over. We hypothesise that in early stages of the pandemic, case detection is higher in countries with older populations due to the higher burden of mortality among older adults [51]. COVID-19 transmission may remain undetected longer in younger populations [78]. Population variable 3 is household size (mean) is the average number of people per dwelling. Individuals in larger households interact with more people including once stay-home measures are applied. For example, analysis of demographic and socioeconomic determinants of COVID-19 testing in New York shows a very strong correlation between infection rate and household size [9]. Population variable 4 is population (n), which is a demographic variable with a direct relation to the pool size for the potentially infected population. Population size was considered as a moderating variable in a previous study [34] that found that “a one person increase in population size indicates over 1.6 more COVID-19 cases” (p. 385) thus more populous countries have greater potential for exposure. Even when normalised on a per capita basis, the likelihood of new cases is still higher in large countries than in small countries. Table 1 lists the variables in the model, with the source, units and year of each.

**Table 1:** List of independent variables to explain the diffusion of reported COVID-19 cases

Variable Description	Category	Units (Transformation)	Source	Year
Interpersonal Globalisation	Globalisation	Index Value (100 Point Scale)	Swiss Economic Institute (KOF)	2019
Trade Globalisation	Globalisation	Index Value (100 Point Scale)	Swiss Economic Institute (KOF)	2019
Financial Globalisation	Globalisation	Index Value (100 Point Scale)	Swiss Economic Institute (KOF)	2019
Urbanisation Rate	Settlement	National (Percent)	World Bank	2018
Population Density	Settlement	Log transformed value of Inhabitants per square kilometer	World Bank	2018
Urban Density	Settlement	Inhabitants per square kilometer in Densest Metropolitan Area	Demographia	2020
Areal Accessibility	Settlement	The area-weighted average for driving time to a location with at least 1500 inhabitants per square kilometer	Weiss et al. (2018)	2018
Human Development	Population	Index Value	United Nations Development Programme	2018
Population aged 65 and over	Population	Percent Age 65+	United Nations, Department of Economic and Social Affairs Population Division	2019
Household Size	Population	Mean Number of Household Members	United Nations, Department of Economic and Social Affairs Population Division	2019
Population	Population	Total population	United Nations	2019

The table below (Table 2) provides summary statistics of the dependent variables by week, as well as independent variables on globalisation, settlement characteristics and population data.

**Table 2:** Descriptive Summary of Variables

Variable	Median	Mean	St. Dev.	Min	Max
Reported cases (per million) by March 4th[log]	0.71	0.83	1.10	−1.29	3.25
Reported cases (per million) by March 11th [log]	1.59	1.60	1.00	−0.83	3.52
Reported cases (per million) by March 18th [log]	2.28	2.23	0.91	−0.01	3.97
Reported cases (per million) by March 25th [log]	2.77	2.69	0.88	0.50	4.29
Reported cases (per million) by April 1st [log]	3.15	3.04	0.86	0.70	4.53
Reported cases (per million) by April 8th [log]	3.38	3.32	0.83	0.89	4.76
Interpersonal Globalisation [index]	68.50	64.80	20.70	22.70	96.50
Trade Globalisation [index]	62.80	57.80	21.50	21.20	99.20
Financial Globalisation [index]	72.70	69.20	19.10	21.30	97.30
Urbanisation [rate]	72.00	68.60	19.60	18.50	100.00
Population Density [log]	1.97	1.95	0.57	0.31	3.90
Urban Density [maximum]	5650	7686	6251	1300	41,000
Areal Accessibility [mean]	111	158	116	30	577
Human Development [index]	0.80	0.79	0.12	0.43	0.95
Aged over 65 [%]	11.00	11.40	6.70	1.09	27.60
Population [million]	18	76	219	1	1432
Household Size [mean]	3.36	3.78	1.16	2.05	8.66

## Results

**Fig. 4:**
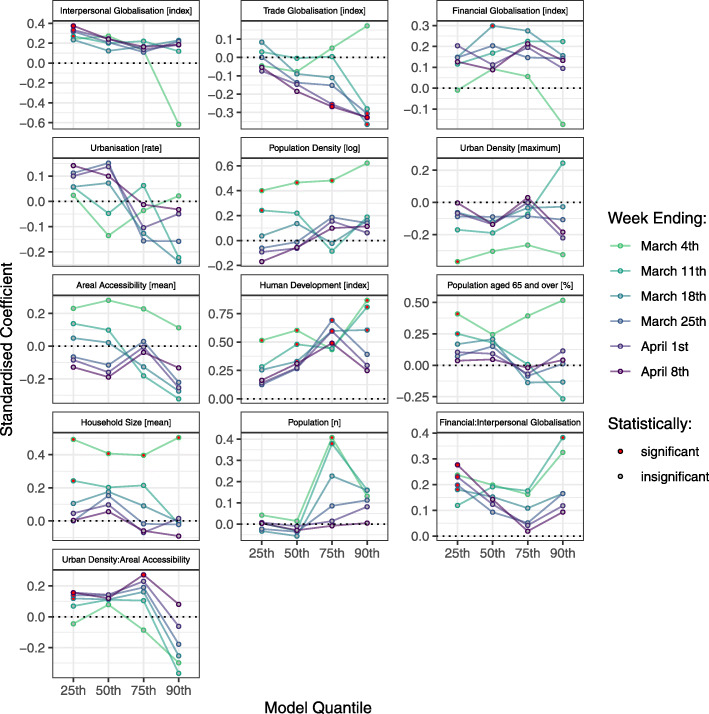
Standardised coefficient value of reported COVID-19 cases at the 25th, 50th, 75th and 90th quantiles the 10th week (ending March 4th) until the 15th week of 2020 (ending April 8th)

Globalisation, settlement characteristics and population characteristics all influence reported COVID-19 diffusion, but do so differently at varied points along the distribution as well as differently across time. Figure 4 visualises the standardised relationship of each factor with the number of (log-transformed) reported cases per million at the 25th, 50th, 75th and 90th quantiles for each of week of the 6 week period.

In the early stages (Weeks 10, 11), population characteristics were the most influential variables in explaining reported COVID-19 diffusion. HDI was found to be the most influential significant variable affecting reported COVID-19 diffusion, particularly in countries with a high number of new cases per capita (75th and 90th quantiles) and within the earlier weeks (supporting earlier findings [34]), decreasing in importance over time. Aged population (65+) is significant only in early weeks at the 25th and 50th quantiles, but strong collinearity with HDI suggests these are related in causality (See Additional file 7). Both HDI and Population aged 65+ tend toward zero in later weeks, indicating declining impact as time goes on. Population size and household size are both positively associated in earlier weeks, which diminishes in later weeks. Population size is significant at the 75th quantile whereas household size is significant throughout the 25th, 50th and 75th quantiles. Population characteristics generally had a declining impact on reported COVID-19 diffusion as the weeks went on.

Settlement characteristics had mixed impacts on reported COVID-19 diffusion. Population density initially (Week 10) had a strong positive influence at the mean, and at the 25th, 50th, and 75th quantiles that waned with time. Maximum urban density exerts negative influence on reported COVID-19 diffusion throughout the distribution, but is strongest at the mean and only significant in the first week of our study. Again, early reported COVID-19 diffusion is tied to density, but the influence of a single (or multiple) densely populated settlements has declining impact over time. In contrast, areal accessibility is negatively associated with reported COVID-19 diffusion in later weeks but only at the 90th quantile, indicating its effect is significant in countries with a high number of new cases per million. A negative relationship suggests that the highest number of total cases are associated with greater access to cities, and that as this is reduced, so are the number of reported cases per million. With the exception of urbanisation, settlement characteristics generally had a generally declining impact on reported COVID-19 diffusion as the weeks went on.

Globalisation has the weakest effect of the three classes of variables, and its effects are mixed both in terms of which portion of the distribution is impacted and the type of globalisation. However, in contrast to the other sets of variables, globalisation had an enhanced (rather than muted) impact on reported COVID-19 diffusion over time (i.e. as the weeks went on).

Interpersonal globalisation has a weak positive effect at the mean and 25th quantile, particularly in early weeks. While financial globalisation was not a reliable predictor, it interacted with interpersonal globalisation towards the start of the study period at both tails of the distribution. Trade globalisation is the most prominent in scaled terms and given that it explains suppressed reported COVID-19 diffusion, suggesting that countries with strong import and export ties are better placed to slow the spread following the closure of borders.

Greater significance in terms of which globalisation and settlement characteristics explain diffusion was added through two interaction terms, added based on goodness-of-fit. The globalisation interaction term is between de facto financial globalisation and de facto interpersonal globalisation. This interaction term takes into account the combined effect of international travel and the level of financial globalisation. This interaction effect is significant and positive, particularly throughout the lower quantiles and in the early weeks. This is to say that countries with a low number of reported COVID-19 cases per million are likely to receive new cases if conditions of both high financial globalisation and interpersonal globalisation are met, generally both related to intensity of human mobility flows.

The settlement interaction term is between urban density of the largest city of the country and the (lack of) accessibility of smaller settlements. This interaction term accounts for the hierarchical connectivity between settlements of different sizes within the country and thus it proxies primacy, as many countries are poorly connected overall but have large and dense capital or primate cities. This interaction yields a mostly positive effect (up to the 75th quantile), and is significant and positive in the distribution in the final week of the analysis.

## Discussion

With the rollout of COVID-19 vaccines still underway on a global scale, the disease continues to be a major detriment to human health. Of the variables examined in our diffusion model, population and settlement characteristics influence new reported COVID-19 cases per one million inhabitants in early weeks while globalisation variables influence new reported COVID-19 cases per one million inhabitants in later weeks. Notably, among countries with a high number of early reported cases, HDI is by far the strongest predictor of new cases. HDI has a strong, albeit weakening, positive association with reported COVID-19 diffusion across the 6 week period, suggesting some level of hierarchical diffusion from more developed countries to less developed countries, and relocation diffusion between more-developed countries with high mobility (e.g. within Europe). However, this could also reflect relatively higher testing and/or reporting levels in affluent countries. Alternatively, there could be relatively larger numbers of asymptomatic cases in less-developed countries on account of typically younger population profiles. As such, we stress that any research using globally aggregated data sets should be interpreted with care.

Particularly in the early weeks, other population and settlement characteristics such as population aged 65+, household size, and population density explain diffusion, but their effect declines in successive weeks. The lasting impact of HDI, and the muted impacts of other population and settlement characteristics, is perhaps best explained by COVID-19’s impacts on mobility. Although more-developed countries may have been more successful in implementing early lock-down measures, they also had much higher overall levels of both international and internal mobility, hence why settlement characteristics play such an important role in the first week of the study (Week 10) but not afterward.

While the impact of settlement and population characteristics generally declines over time, globalisation shows an increased importance in predicting reported COVID-19 diffusion, through this has both negative (trade globalisation) and positive (interpersonal globalisation) effects. Of the globalisation variables, interpersonal globalisation has the strongest effect, particularly when interacting with the financial globalisation variable. This suggests that continued human mobility may be a critical determinant of reported COVID-19 diffusion.

Conversely, trade globalisation has a negative impact, and the impacts of all three globalisation types appear to be stronger toward the latter weeks. The impact of globalisation in later weeks is somewhat counterintuitive if one expects more globalised countries to experience rapid COVID-19 diffusion in earlier stages and other countries to reach similar levels over time. It also reflects the fact that the economies of more globalised countries are tied to ‘openness’, with strong disincentives for shutting borders and enforcing other ‘global’ restrictions. To this end, trade globalisation is not associated with human mobility as much as financial globalisation and interpersonal globalisation, with the latter incorporating both tourism and migration.

## Conclusion

Globalisation, settlement characteristics and population characteristics are all important in explaining reported COVID-19 diffusion, but significant at different points on the distribution and at different points in time. Population and settlement characteristics were most influential in explaining COVID-19 diffusion in the weeks surrounding the WHO declaring the global pandemic in March 2020, but in subsequent weeks globalisation became more important. This exploratory analysis suggests that both hierarchical and relocation diffusion were responsible for reported disease spread, as more globalised and developed countries (measured by HDI) spread COVID-19 to less globalised and/or developed countries, and that this process was accelerated early-on in countries with high urban density and accessibility.

The model reveals that urbanisation and density generally exert a positive effect on disease diffusion early-on, but that over time this impact tends toward zero. Conversely, variants of globalisation exert diverse effects, with trade globalisation exerting a negative effect on reported COVID-19 diffusion that diverges from the positive effects associated with financial and interpersonal globalisation. Our quantile regression modelling approach highlights that impacts of settlement characteristics are mixed but generally exert the greatest impact towards the lower and higher quantiles, and particularly in the earlier weeks.

Our findings suggest that the impacts of non-local diffusion outweigh the geographical effects of diffusion tied to adjacency, at least early on in the pandemic. Although both infectious and contagious diffusions are present throughout the study period via interpersonal contact, our results indicate that relocation diffusion precedes hierarchical diffusion as the disease is first spread within affluent and mobile countries, then carried across long distances via global mobility, and later diffused within countries from single or multiple points of entry, which are typically the largest and/or most globalised cities. Though this may seem self-evident, further research should focus on the impacts and effects of policy on diffusion, which is likely to have had a strong impact across the study period [79–81].

Perhaps the finding that more-developed countries experience higher disease diffusion before less-developed countries may be perceived as auspicious, given that countries with better governance, more economic wealth and more advanced health care systems are better able to cope with pandemic conditions. It may, however, reflect differences in reporting standards and/or testing rates, which would be highest in affluent countries early-on.

Our model finds clear evidence of diffusion: from more-developed to less-developed; and to a lesser extent from urbanised to non-urbanised. As COVID-19 is a disease whose diffusion is reliant on interpersonal transmission, we find that both relocation diffusion (tied to global mobility) and hierarchical diffusion (tied to population and settlement characteristics) are simultaneously acting on countries.

To date, the primary mobility-focused public health initiatives to curb disease diffusion have been travel bans (border closures) and stay-home orders that restrict gatherings. Both have shown clear effectiveness in curbing disease diffusion [79, 81] as both Australia and New Zealand all but vanquishing COVID-19 has proven [82]. As disease diffusion progresses, implementing these measures at increasingly small scales will be necessary as restricting human mobility has proven the most effective measure against the spread.

With vaccines on the horizon alongside increasing impatience around ‘returning to normal’, the efficacy of government mandates in many contexts comes into question [83–86]. One consideration is to weigh the implications of a curative (e.g. vaccines) versus preventative (e.g. distancing, restricted mobility) approach to the pandemic. To this end, as we know that globalisation through interpersonal mobility is a contributor to disease spread, international and interregional travel may be worth limiting, or arresting entirely if the latter approach is pursued. By the same token, spatial analysis suggests that targeted epidemiological interventions may be most effective, which may in fact combine strategies. As our data have shown, certain settlement and population characteristics create the preconditions for reported COVID-19 diffusion, yet these are far more difficult to modify (e.g. de-urbanisation, de-densification) than it is to reduce globally high levels of mobility. Governments must enlist the efforts of social scientists to better understand how spatially targeted interventions can curb disease diffusion, and by corollary transmission.

## Methods

An Ordinary Least Squares regression (OLS; formula 1) was repeated for each period (weeks 10 to 15). We introduce two interaction terms - one at the global scale and another at the local-scale. At the global scale, the interaction term is between de facto financial and interpersonal globalisation. Financial globalisation captures direct foreign investment, international reserves, and international income payments that induce movement of skills and labour. Financially globalised nations are typically global centres of business and related services and thus, generate global business travel and interaction. As such, the interaction between financial and interpersonal globalisation captures international travel related to business. In contrast, we anticipate that the national-scale interaction between maximum urban density (the largest National City) and areal accessibility will have growing importance in later weeks once national borders close and thus COVID-19 exposure will typically occur within national borders and at home. As such, this interaction represents the connectivity between the smaller urban growth centres and the economic centre of the country.

1 $$ {y}_i={\beta}_0+{\beta}_1{x}_1+{\beta}_2{x}_2+\dots +{\beta}_n{x}_n+{\varepsilon}_n $$

where *y*_*n*_ is the log-transformed rate of reported COVID-19 cases, *β*_0_ is the y-intercept, *β*_*n*_*x*_*n*_ are coefficients for the explanatory variables, and *ε*_*n*_ is the error term.

Once the least parsimonious and collinear set of explanatory variables was identified using an empirical LASSO method that iterates through all combinations of globalisation and national explanatory variables, quantile regression was used to explain the global diffusion and transmission of COVID-19 at specific points along the distribution using these explanatory variables (see Additional file 7). This regression revealed how the influences of log-transformed rate of reported COVID-19 cases vary across the quantiles of the distribution [87]. As such, this regression does not assume there is normality nor uniformity in how COVID-19 is diffused and transmitted between and within countries. This regression revealed how the influences of log-transformed rate of reported COVID-19 cases vary across the quantiles of the distribution [87]. As such, this regression does not assume there is normality nor uniformity in how COVID-19 is diffused and transmitted between and within countries. The τ were placed at the 25th, 50th, 75th, and 90th quartiles according to the conventions of disease mapping [88–90]. Again the quantile regression was iterated for each week using formula 2 [87]:

2 $$ {Q}^{\uptau}\left({y}_i|{x}_i\right)={\beta}_0^{\left(\uptau \right)}+{\beta}_1^{\left(\uptau \right)}{x}_1+\dots +{\beta}_n^{\left(\uptau \right)}{x}_n+{\varepsilon}^{\left(\tau \right)} $$

where *Q*^τ^ is a point estimate for *y*_*i*_ given *x*_*i*_, and where τ is specific quantiles (i.e. the 25th, 50th, 75th, and 90th), *y*_*i*_ is the log-transformed rate of reported COVID-19 cases for country *i* and *x*_*i*_ are explanatory variables. On the explanatory-side of formula, ${\beta}_0^{\left(\uptau \right)}$ are y-intercepts, ${\beta}_n^{\left(\uptau \right)}{x}_n$ coefficients for the explanatory variables, and *ε*^(*τ*)^ are error terms for each quantile τ.

The output tables for these regression models are provided in Additional files 1, 2, 3, 4, 5 and 6. Lastly, the specific R function used for modelling is quantreg::rq for quantile regression.

Koenker and Machado (1999) suggest a goodness of fit, R_1_ (τ) analogous to R-squared in simple linear regression and argues that R_1_ (τ) gives a local measure of goodness of fit for a particular quantile rather than a global measure of goodness of fit over the entire conditional distribution [91]. The median (50th quantile) is the point at which the model is weakest, suggesting likewise that a mean model would have been a poor fit. The model is strongest at the 25th and 90th quantiles, indicating that the model is best fit to serve countries with a low number of cases (these are mostly small countries with low HDI) and the 90th is where most of the existing cases are (generally larger countries with high HDI). The quantile regression model is the best fit in the first week, with progressively less significance and explanatory power. This suggests that policy may be most effective in early weeks, as known socio-spatial conditions can be targeted through specific public interventions.

## Supplementary Information

**Additional file 1.** Week 10 (ending April 4th) comparison of standardised coefficients at 25th, 50th, 75th and 90th quantiles and the mean function.

**Additional file 2.** Week 11 (ending March 11th) comparison of standardised coefficients at 25th, 50th, 75th and 90th quantiles and the mean function.

**Additional file 3.** Week 12 (ending March 18th) comparison of standardised coefficients at 25th, 50th, 75th and 90th quantiles and the mean function.

**Additional file 4.** Week 13 (ending March 25th) comparison of standardised coefficients at 25th, 50th, 75th and 90th quantiles and the mean function.

**Additional file 5.** Week 14 (ending April 1st) comparison of standardised coefficients at 25th, 50th, 75th and 90th quantiles and the mean function.

**Additional file 6.** Week 15 (ending April 8th) comparison of standardised coefficients at 25th, 50th, 75th and 90th quantiles and the mean function.

**Additional file 7.** Correlogram and Multicollinearity Diagnostics.

## Data Availability

The dataset supporting the conclusions of this article is available from the COVID-19 Data Repository by the Center for Systems Science and Engineering (CSSE) at Johns Hopkins University, https://github.com/CSSEGISandData/COVID-19.
