# Supplementary material for: The socio-spatial determinants of COVID-19 diffusion: the impact of globalisation, settlement characteristics and population
Source: Global Health. 2021 May 20;17:56. doi: 10.1186/s12992-021-00707-2 (PMC8135172; doi:10.1186/s12992-021-00707-2)
Supplement: Supplementary file 1 — Additional file 1. Week 10 (ending April 4th) comparison of standardised coefficients at 25th, 50th, 75th and 90th quantiles and the mean function. [file 12992_2021_707_MOESM1_ESM.docx]

# **Additional file 1.** **Week 10 (ending April 4th) comparison of standardised coefficients at 25th, 50th, 75th and 90th quantiles and the mean function**

|  | | | | | |
| --- | --- | --- | --- | --- | --- |
|  | Dependent variable: | | | | |
|  |  | | | | |
|  | OLS | quantile | | | |
|  |  | regression | | | |
|  | Mean Model | 25th quantile | 50th quantile | 75th quantile | 90th quantile |
|  | | | | | |
| Intercept | 0.663^***^ | 0.257 | 0.644^***^ | 1.070^***^ | 1.490^***^ |
|  | (0.108) | (0.162) | (0.171) | (0.190) | (0.195) |
| Interpersonal Globalisation [index] | 0.134 | 0.362 | 0.227 | 0.149 | -0.503 |
|  | (0.171) | (0.238) | (0.254) | (0.225) | (0.419) |
| Trade Globalisation [index] | 0.082 | -0.008 | 0.074 | 0.116 | 0.099 |
|  | (0.127) | (0.152) | (0.185) | (0.205) | (0.239) |
| Financial Globalisation [index] | -0.134 | -0.082 | 0.073 | 0.040 | -0.041 |
|  | (0.159) | (0.237) | (0.245) | (0.231) | (0.262) |
| Urbanisation [rate] | -0.024 | 0.086 | 0.030 | -0.071 | -0.065 |
|  | (0.129) | (0.162) | (0.180) | (0.203) | (0.264) |
| Population Density [log] | 0.505^***^ | 0.441^**^ | 0.416^**^ | 0.408 | 0.519 |
|  | (0.156) | (0.185) | (0.204) | (0.247) | (0.349) |
| Urban Density [maximum] | -0.357^**^ | -0.383^**^ | -0.191 | -0.133 | -0.145 |
|  | (0.147) | (0.163) | (0.202) | (0.495) | (0.689) |
| Areal Accessibility [mean] | 0.311^**^ | 0.300 | 0.335 | 0.218 | -0.015 |
|  | (0.155) | (0.197) | (0.229) | (0.367) | (0.416) |
| Human Development [index] | 0.636^***^ | 0.396 | 0.462 | 0.477 | 0.959^**^ |
|  | (0.210) | (0.290) | (0.322) | (0.337) | (0.394) |
| Population aged 65 and over [%] | 0.337^*^ | 0.344 | 0.408^*^ | 0.393 | 0.237 |
|  | (0.179) | (0.220) | (0.238) | (0.271) | (0.365) |
| Household Size [mean] | 0.344^**^ | 0.356^*^ | 0.442^**^ | 0.384^**^ | 0.402 |
|  | (0.144) | (0.208) | (0.194) | (0.179) | (0.277) |
| Population [n] | 0.165^*^ | 0.061 | 0.039 | 0.415^**^ | 0.156 |
|  | (0.091) | (0.151) | (0.188) | (0.173) | (0.213) |
| Financial:Interpersonal Globalisation | 0.211^**^ | 0.284^*^ | 0.129 | 0.169 | 0.354 |
|  | (0.102) | (0.145) | (0.153) | (0.177) | (0.229) |
| Urban Density:Areal Accessibility | 0.048 | -0.027 | 0.074 | -0.136 | -0.343 |
|  | (0.090) | (0.112) | (0.130) | (0.377) | (0.452) |
|  | | | | | |
| Observations | 84 | 84 | 84 | 84 | 84 |
| R^2^ | 0.678 |  |  |  |  |
| Adjusted R^2^ | 0.619 |  |  |  |  |
| Residual Std. Error | 0.679 |  |  |  |  |
| F Statistic | 11.400^***^ |  |  |  |  |
|  | | | | | |
| Note: | ^*^p^**^p^***^p<0.01 | | | | |
