# Supplementary material for: The socio-spatial determinants of COVID-19 diffusion: the impact of globalisation, settlement characteristics and population
Source: Global Health. 2021 May 20;17:56. doi: 10.1186/s12992-021-00707-2 (PMC8135172; doi:10.1186/s12992-021-00707-2)
Supplement: Supplementary file 2 — Additional file 2. Week 11 (ending March 11th) comparison of standardised coefficients at 25th, 50th, 75th and 90th quantiles and the mean function. [file 12992_2021_707_MOESM2_ESM.docx]

# **Additional file 2. Week 11 (ending March 11th) comparison of standardised coefficients at 25th, 50th, 75th and 90th quantiles and the mean function**

|  | | | | | |
| --- | --- | --- | --- | --- | --- |
|  | Dependent variable: | | | | |
|  |  | | | | |
|  | OLS | quantile | | | |
|  |  | regression | | | |
|  | Mean Model | 25th quantile | 50th quantile | 75th quantile | 90th quantile |
|  | | | | | |
| Intercept | 1.460^***^ | 1.210^***^ | 1.440^***^ | 1.790^***^ | 2.100^***^ |
|  | (0.089) | (0.101) | (0.121) | (0.148) | (0.174) |
| Interpersonal Globalisation [index] | 0.104 | 0.251^*^ | 0.110 | 0.031 | -0.033 |
|  | (0.141) | (0.145) | (0.149) | (0.201) | (0.316) |
| Trade Globalisation [index] | 0.049 | 0.033 | 0.047 | 0.058 | -0.161 |
|  | (0.105) | (0.111) | (0.125) | (0.170) | (0.208) |
| Financial Globalisation [index] | 0.030 | 0.141 | 0.171 | 0.234 | 0.121 |
|  | (0.131) | (0.146) | (0.155) | (0.168) | (0.212) |
| Urbanisation [rate] | -0.014 | 0.077 | 0.072 | -0.012 | -0.184 |
|  | (0.106) | (0.104) | (0.116) | (0.161) | (0.238) |
| Population Density [log] | 0.262^**^ | 0.223^*^ | 0.271 | 0.273 | 0.342 |
|  | (0.129) | (0.121) | (0.170) | (0.250) | (0.301) |
| Urban Density [maximum] | -0.205^*^ | -0.171 | -0.130 | -0.123 | 0.059 |
|  | (0.121) | (0.107) | (0.124) | (0.242) | (0.599) |
| Areal Accessibility [mean] | 0.141 | 0.123 | 0.127 | 0.101 | -0.149 |
|  | (0.128) | (0.131) | (0.156) | (0.240) | (0.336) |
| Human Development [index] | 0.629^***^ | 0.261 | 0.393^*^ | 0.481^*^ | 0.786^**^ |
|  | (0.174) | (0.182) | (0.199) | (0.255) | (0.304) |
| Population aged 65 and over [%] | 0.235 | 0.269^**^ | 0.354^*^ | 0.388 | 0.114 |
|  | (0.148) | (0.133) | (0.195) | (0.282) | (0.332) |
| Household Size [mean] | 0.292^**^ | 0.274^**^ | 0.332^**^ | 0.338^**^ | 0.183 |
|  | (0.118) | (0.110) | (0.135) | (0.167) | (0.225) |
| Population [n] | 0.100 | -0.001 | -0.023 | 0.310^**^ | 0.137 |
|  | (0.075) | (0.113) | (0.122) | (0.131) | (0.162) |
| Financial:Interpersonal Globalisation | 0.160^*^ | 0.160 | 0.108 | 0.102 | 0.367^*^ |
|  | (0.084) | (0.101) | (0.113) | (0.145) | (0.211) |
| Urban Density:Areal Accessibility | 0.122 | 0.075 | 0.122 | 0.044 | -0.308 |
|  | (0.075) | (0.072) | (0.081) | (0.194) | (0.405) |
|  | | | | | |
| Observations | 84 | 84 | 84 | 84 | 84 |
| R^2^ | 0.735 |  |  |  |  |
| Adjusted R^2^ | 0.686 |  |  |  |  |
| Residual Std. Error | 0.560 |  |  |  |  |
| F Statistic | 14.900^***^ |  |  |  |  |
|  | | | | | |
| Note: | ^*^p^**^p^***^p<0.01 | | | | |
