# Supplementary material for: The socio-spatial determinants of COVID-19 diffusion: the impact of globalisation, settlement characteristics and population
Source: Global Health. 2021 May 20;17:56. doi: 10.1186/s12992-021-00707-2 (PMC8135172; doi:10.1186/s12992-021-00707-2)
Supplement: Supplementary file 3 — Additional file 3. Week 12 (ending March 18th) comparison of standardised coefficients at 25th, 50th, 75th and 90th quantiles and the mean function. [file 12992_2021_707_MOESM3_ESM.docx]

# **Additional file 3. Week 12 (ending March 18th) comparison of standardised coefficients at 25th, 50th, 75th and 90th quantiles and the mean function**

|  | | | | | |
| --- | --- | --- | --- | --- | --- |
|  | Dependent variable: | | | | |
|  |  | | | | |
|  | OLS | quantile | | | |
|  |  | regression | | | |
|  | Mean Model | 25th quantile | 50th quantile | 75th quantile | 90th quantile |
|  | | | | | |
| Intercept | 2.100^***^ | 1.780^***^ | 2.100^***^ | 2.380^***^ | 2.840^***^ |
|  | (0.082) | (0.113) | (0.123) | (0.151) | (0.162) |
| Interpersonal Globalisation [index] | 0.140 | 0.170 | 0.139 | 0.150 | 0.114 |
|  | (0.130) | (0.140) | (0.156) | (0.227) | (0.286) |
| Trade Globalisation [index] | -0.024 | 0.049 | -0.105 | -0.138 | -0.379^*^ |
|  | (0.096) | (0.116) | (0.116) | (0.162) | (0.199) |
| Financial Globalisation [index] | 0.113 | 0.236 | 0.198 | 0.232 | 0.165 |
|  | (0.120) | (0.157) | (0.152) | (0.188) | (0.184) |
| Urbanisation [rate] | 0.023 | 0.086 | 0.035 | -0.187 | -0.242 |
|  | (0.098) | (0.106) | (0.112) | (0.193) | (0.215) |
| Population Density [log] | 0.105 | 0.025 | 0.161 | 0.168 | 0.227 |
|  | (0.118) | (0.128) | (0.159) | (0.249) | (0.265) |
| Urban Density [maximum] | -0.149 | -0.067 | -0.167 | -0.090 | -0.137 |
|  | (0.111) | (0.115) | (0.113) | (0.221) | (0.437) |
| Areal Accessibility [mean] | 0.004 | 0.008 | 0.037 | 0.033 | -0.174 |
|  | (0.118) | (0.131) | (0.138) | (0.257) | (0.290) |
| Human Development [index] | 0.485^***^ | 0.229 | 0.336^*^ | 0.616^**^ | 0.646^**^ |
|  | (0.160) | (0.189) | (0.193) | (0.295) | (0.280) |
| Population aged 65 and over [%] | 0.108 | 0.226 | 0.291 | -0.005 | -0.001 |
|  | (0.136) | (0.151) | (0.191) | (0.279) | (0.292) |
| Household Size [mean] | 0.146 | 0.180 | 0.265^*^ | 0.119 | 0.083 |
|  | (0.109) | (0.125) | (0.142) | (0.180) | (0.194) |
| Population [n] | 0.060 | -0.041 | -0.059 | 0.185 | 0.143 |
|  | (0.069) | (0.119) | (0.111) | (0.154) | (0.155) |
| Financial:Interpersonal Globalisation | 0.142^*^ | 0.199^*^ | 0.101 | 0.091 | 0.105 |
|  | (0.077) | (0.104) | (0.115) | (0.149) | (0.162) |
| Urban Density:Areal Accessibility | 0.138^**^ | 0.104 | 0.098 | 0.171 | -0.249 |
|  | (0.069) | (0.072) | (0.071) | (0.212) | (0.315) |
|  | | | | | |
| Observations | 84 | 84 | 84 | 84 | 84 |
| R^2^ | 0.729 |  |  |  |  |
| Adjusted R^2^ | 0.679 |  |  |  |  |
| Residual Std. Error | 0.515 |  |  |  |  |
| F Statistic | 14.500^***^ |  |  |  |  |
|  | | | | | |
| Note: | ^*^p^**^p^***^p<0.01 | | | | |
