# Supplementary material for: The socio-spatial determinants of COVID-19 diffusion: the impact of globalisation, settlement characteristics and population
Source: Global Health. 2021 May 20;17:56. doi: 10.1186/s12992-021-00707-2 (PMC8135172; doi:10.1186/s12992-021-00707-2)
Supplement: Supplementary file 4 — Additional file 4. Week 13 (ending March 25th) comparison of standardised coefficients at 25th, 50th, 75th and 90th quantiles and the mean function. [file 12992_2021_707_MOESM4_ESM.docx]

# **Additional file 4. Week 13 (ending March 25th) comparison of standardised coefficients at 25th, 50th, 75th and 90th quantiles and the mean function**

|  | | | | | |
| --- | --- | --- | --- | --- | --- |
|  | Dependent variable: | | | | |
|  |  | | | | |
|  | OLS | quantile | | | |
|  |  | regression | | | |
|  | Mean Model | 25th quantile | 50th quantile | 75th quantile | 90th quantile |
|  | | | | | |
| Intercept | 2.580^***^ | 2.270^***^ | 2.590^***^ | 2.930^***^ | 3.190^***^ |
|  | (0.077) | (0.124) | (0.144) | (0.141) | (0.124) |
| Interpersonal Globalisation [index] | 0.184 | 0.240 | 0.172 | 0.184 | 0.097 |
|  | (0.122) | (0.158) | (0.186) | (0.216) | (0.231) |
| Trade Globalisation [index] | -0.072 | -0.034 | -0.095 | -0.248 | -0.311^*^ |
|  | (0.091) | (0.116) | (0.138) | (0.151) | (0.157) |
| Financial Globalisation [index] | 0.141 | 0.244 | 0.168 | 0.223 | 0.148 |
|  | (0.114) | (0.161) | (0.182) | (0.185) | (0.159) |
| Urbanisation [rate] | 0.053 | 0.040 | 0.110 | -0.176 | -0.148 |
|  | (0.092) | (0.101) | (0.131) | (0.195) | (0.188) |
| Population Density [log] | -0.009 | -0.024 | 0.008 | 0.322 | 0.191 |
|  | (0.112) | (0.142) | (0.192) | (0.244) | (0.213) |
| Urban Density [maximum] | -0.112 | -0.053 | -0.139 | -0.039 | -0.224 |
|  | (0.105) | (0.110) | (0.132) | (0.248) | (0.329) |
| Areal Accessibility [mean] | -0.096 | -0.029 | -0.121 | 0.150 | -0.138 |
|  | (0.111) | (0.132) | (0.167) | (0.307) | (0.227) |
| Human Development [index] | 0.408^***^ | 0.235 | 0.226 | 0.562^*^ | 0.432^*^ |
|  | (0.151) | (0.183) | (0.223) | (0.285) | (0.241) |
| Population aged 65 and over [%] | 0.037 | 0.219 | 0.178 | 0.169 | 0.144 |
|  | (0.128) | (0.170) | (0.235) | (0.221) | (0.233) |
| Household Size [mean] | 0.070 | 0.141 | 0.102 | 0.134 | 0.077 |
|  | (0.103) | (0.142) | (0.172) | (0.162) | (0.157) |
| Population [n] | 0.044 | -0.030 | -0.048 | 0.070 | 0.104 |
|  | (0.065) | (0.108) | (0.134) | (0.165) | (0.128) |
| Financial:Interpersonal Globalisation | 0.130^*^ | 0.191^*^ | 0.093 | 0.016 | 0.102 |
|  | (0.073) | (0.111) | (0.133) | (0.157) | (0.135) |
| Urban Density:Areal Accessibility | 0.151^**^ | 0.134^**^ | 0.128 | 0.209 | -0.182 |
|  | (0.065) | (0.065) | (0.084) | (0.321) | (0.250) |
|  | | | | | |
| Observations | 84 | 84 | 84 | 84 | 84 |
| R^2^ | 0.741 |  |  |  |  |
| Adjusted R^2^ | 0.693 |  |  |  |  |
| Residual Std. Error | 0.487 |  |  |  |  |
| F Statistic | 15.400^***^ |  |  |  |  |
|  | | | | | |
| Note: | ^*^p^**^p^***^p<0.01 | | | | |
