# Supplementary material for: The socio-spatial determinants of COVID-19 diffusion: the impact of globalisation, settlement characteristics and population
Source: Global Health. 2021 May 20;17:56. doi: 10.1186/s12992-021-00707-2 (PMC8135172; doi:10.1186/s12992-021-00707-2)
Supplement: Supplementary file 5 — Additional file 5. Week 14 (ending April 1st) comparison of standardised coefficients at 25th, 50th, 75th and 90th quantiles and the mean function. [file 12992_2021_707_MOESM5_ESM.docx]

# **Additional file 5. Week 14 (ending April 1st) comparison of standardised coefficients at 25th, 50th, 75th and 90th quantiles and the mean function**

|  | | | | | |
| --- | --- | --- | --- | --- | --- |
|  | Dependent variable: | | | | |
|  |  | | | | |
|  | OLS | quantile | | | |
|  |  | regression | | | |
|  | Mean Model | 25th quantile | 50th quantile | 75th quantile |  |
|  | | | | | |
| Intercept | 2.920^***^ | 2.600^***^ | 2.950^***^ | 3.270^***^ | 3.450^***^ |
|  | (0.073) | (0.128) | (0.149) | (0.127) | (0.113) |
| Interpersonal Globalisation [index] | 0.220^*^ | 0.299^*^ | 0.180 | 0.155 | 0.131 |
|  | (0.116) | (0.169) | (0.195) | (0.179) | (0.207) |
| Trade Globalisation [index] | -0.089 | -0.074 | -0.154 | -0.297^**^ | -0.307^**^ |
|  | (0.086) | (0.121) | (0.137) | (0.141) | (0.140) |
| Financial Globalisation [index] | 0.142 | 0.247 | 0.076 | 0.266 | 0.076 |
|  | (0.108) | (0.167) | (0.191) | (0.174) | (0.151) |
| Urbanisation [rate] | 0.078 | 0.062 | 0.046 | -0.059 | -0.069 |
|  | (0.088) | (0.109) | (0.161) | (0.182) | (0.179) |
| Population Density [log] | -0.101 | -0.076 | 0.013 | 0.177 | 0.099 |
|  | (0.106) | (0.148) | (0.203) | (0.227) | (0.196) |
| Urban Density [maximum] | -0.079 | -0.035 | -0.187 | 0.002 | -0.271 |
|  | (0.100) | (0.120) | (0.149) | (0.254) | (0.288) |
| Areal Accessibility [mean] | -0.172 | -0.079 | -0.118 | -0.028 | -0.192 |
|  | (0.105) | (0.141) | (0.169) | (0.362) | (0.209) |
| Human Development [index] | 0.364^**^ | 0.199 | 0.363 | 0.458 | 0.313 |
|  | (0.143) | (0.193) | (0.258) | (0.277) | (0.228) |
| Population aged 65 and over [%] | -0.018 | 0.167 | 0.143 | 0.075 | 0.186 |
|  | (0.122) | (0.174) | (0.240) | (0.207) | (0.214) |
| Household Size [mean] | 0.023 | 0.139 | 0.096 | 0.029 | 0.057 |
|  | (0.098) | (0.141) | (0.186) | (0.142) | (0.141) |
| Population [n] | 0.044 | 0.006 | -0.024 | 0.033 | 0.067 |
|  | (0.062) | (0.119) | (0.135) | (0.158) | (0.123) |
| Financial:Interpersonal Globalisation | 0.136^*^ | 0.200^*^ | 0.093 | 0.042 | 0.116 |
|  | (0.069) | (0.119) | (0.135) | (0.199) | (0.113) |
| Urban Density:Areal Accessibility | 0.168^***^ | 0.157^**^ | 0.132 | 0.188 | -0.066 |
|  | (0.061) | (0.072) | (0.086) | (0.455) | (0.212) |
|  | | | | | |
| Observations | 84 | 84 | 84 | 84 | 84 |
| R^2^ | 0.755 |  |  |  |  |
| Adjusted R^2^ | 0.710 |  |  |  |  |
| Residual Std. Error | 0.462 |  |  |  |  |
| F Statistic | 16.600^***^ |  |  |  |  |
|  | | | | | |
| Note: | ^*^p^**^p^***^p<0.01 | | | | |
