# Supplementary material for: The socio-spatial determinants of COVID-19 diffusion: the impact of globalisation, settlement characteristics and population
Source: Global Health. 2021 May 20;17:56. doi: 10.1186/s12992-021-00707-2 (PMC8135172; doi:10.1186/s12992-021-00707-2)
Supplement: Supplementary file 6 — Additional file 6. Week 15 (ending April 8th) comparison of standardised coefficients at 25th, 50th, 75th and 90th quantiles and the mean function. [file 12992_2021_707_MOESM6_ESM.docx]

# **Additional file 6. Week 15 (ending April 8th) comparison of standardised coefficients at 25th, 50th, 75th and 90th quantiles and the mean function**

|  | | | | | |
| --- | --- | --- | --- | --- | --- |
|  | Dependent variable: | | | | |
|  |  | | | | |
|  | OLS | quantile | | | |
|  |  | regression | | | |
|  | Mean Model | 25th quantile | 50th quantile | 75th quantile | 90th quantile |
|  | | | | | |
| Intercept | 3.200^***^ | 2.810^***^ | 3.160^***^ | 3.530^***^ | 3.650^***^ |
|  | (0.070) | (0.116) | (0.137) | (0.111) | (0.104) |
| Interpersonal Globalisation [index] | 0.241^**^ | 0.368^**^ | 0.221 | 0.194 | 0.146 |
|  | (0.111) | (0.149) | (0.178) | (0.155) | (0.187) |
| Trade Globalisation [index] | -0.095 | -0.065 | -0.154 | -0.279^**^ | -0.331^***^ |
|  | (0.082) | (0.115) | (0.123) | (0.109) | (0.124) |
| Financial Globalisation [index] | 0.148 | 0.141 | 0.095 | 0.239 | 0.125 |
|  | (0.103) | (0.171) | (0.165) | (0.145) | (0.141) |
| Urbanisation [rate] | 0.098 | 0.138 | 0.073 | 0.003 | 0.015 |
|  | (0.084) | (0.104) | (0.144) | (0.163) | (0.163) |
| Population Density [log] | -0.172^*^ | -0.166 | -0.019 | 0.094 | -0.010 |
|  | (0.101) | (0.121) | (0.183) | (0.160) | (0.172) |
| Urban Density [maximum] | -0.011 | -0.005 | -0.157 | 0.036 | -0.174 |
|  | (0.095) | (0.114) | (0.136) | (0.210) | (0.172) |
| Areal Accessibility [mean] | -0.238^**^ | -0.130 | -0.142 | -0.041 | -0.275^*^ |
|  | (0.100) | (0.130) | (0.149) | (0.173) | (0.162) |
| Human Development [index] | 0.360^**^ | 0.169 | 0.307 | 0.449^*^ | 0.290 |
|  | (0.136) | (0.192) | (0.232) | (0.240) | (0.221) |
| Population aged 65 and over [%] | -0.071 | 0.039 | 0.087 | 0.009 | -0.030 |
|  | (0.116) | (0.147) | (0.215) | (0.192) | (0.186) |
| Household Size [mean] | -0.005 | 0.012 | 0.066 | -0.017 | -0.079 |
|  | (0.093) | (0.128) | (0.164) | (0.125) | (0.129) |
| Population [n] | 0.034 | 0.002 | -0.031 | 0.004 | 0.016 |
|  | (0.059) | (0.111) | (0.119) | (0.067) | (0.102) |
| Financial:Interpersonal Globalisation | 0.134^**^ | 0.278^***^ | 0.155 | 0.012 | 0.106 |
|  | (0.066) | (0.100) | (0.121) | (0.097) | (0.090) |
| Urban Density:Areal Accessibility | 0.169^***^ | 0.154^**^ | 0.104 | 0.276^*^ | 0.110 |
|  | (0.059) | (0.066) | (0.075) | (0.148) | (0.112) |
|  | | | | | |
| Observations | 84 | 84 | 84 | 84 | 84 |
| R^2^ | 0.766 |  |  |  |  |
| Adjusted R^2^ | 0.722 |  |  |  |  |
| Residual Std. Error | 0.440 |  |  |  |  |
| F Statistic | 17.600^***^ |  |  |  |  |
|  | | | | | |
| Note: | ^*^p^**^p^***^p<0.01 | | | | |
