# Supplementary material for: The socio-spatial determinants of COVID-19 diffusion: the impact of globalisation, settlement characteristics and population
Source: Global Health. 2021 May 20;17:56. doi: 10.1186/s12992-021-00707-2 (PMC8135172; doi:10.1186/s12992-021-00707-2)
Supplement: Supplementary file 7 — Additional file 7. Correlogram and Multicollinearity Diagnostics. [file 12992_2021_707_MOESM7_ESM.docx]

**Additional file 7. Correlogram and Multicollinearity Diagnostics**


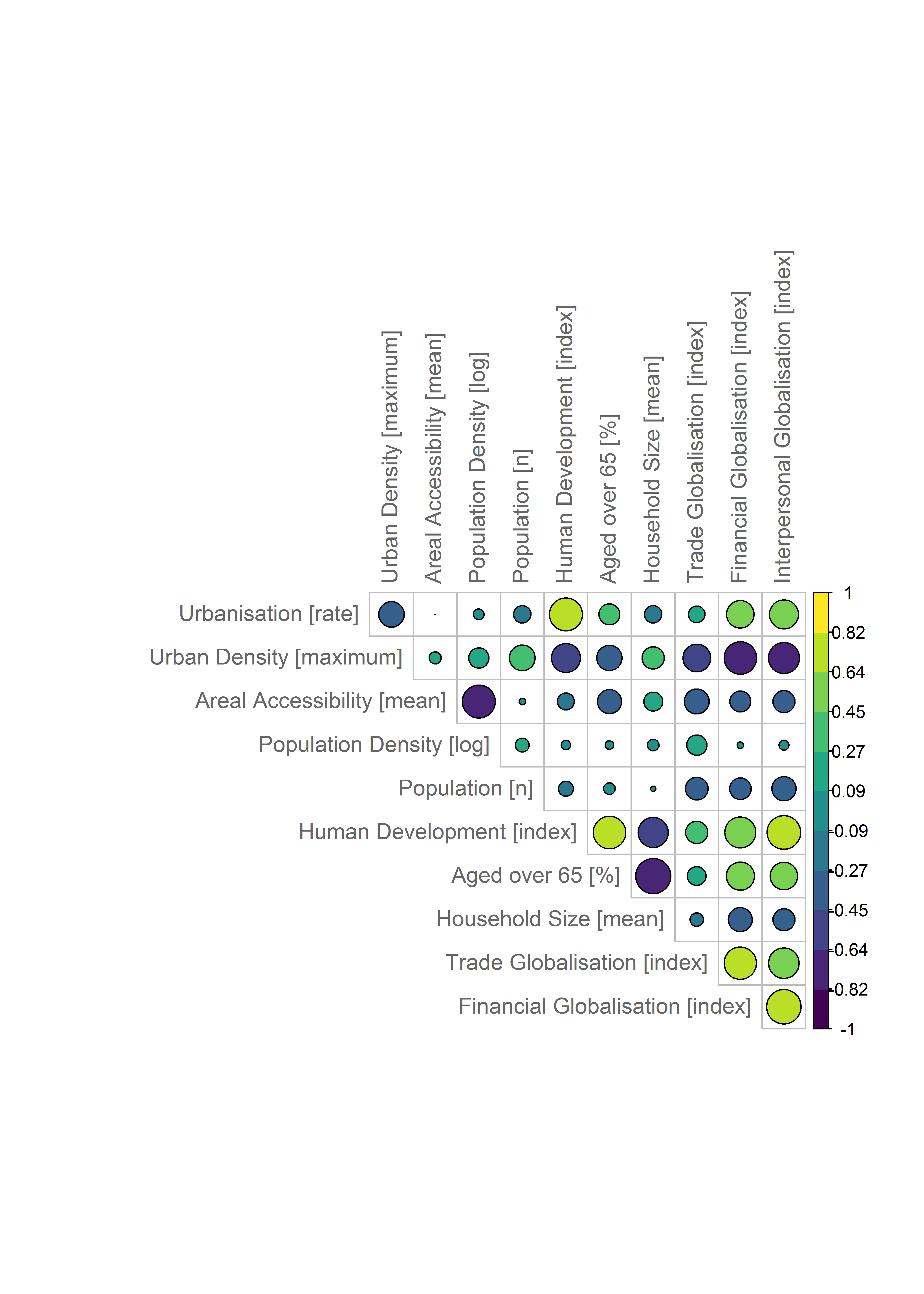


This depicts collinearities among the independent variables that is particularly pronounced between the human development index and globalization indices.
